# Cyclo (Pro-Tyr) upregulates *GmPOD53L* to enhance soybean resistance to cyst nematode (*Heterodera glycines* Ichinohe)

**DOI:** 10.3389/fpls.2025.1628555

**Published:** 2025-07-23

**Authors:** Hui Wang, Yuanjie Li, Xudong Wang, Shumei Liu, Fengjiao Fan, Songjie Qi, Min Wang, Yubo Jia, Qiumin Chen, Yuxi Duan, Chen Liu

**Affiliations:** ^1^ College of Bioscience and Biotechnology, Shenyang Agricultural University, Shenyang, Liaoning, China; ^2^ Key Laboratory of Potato Genetic Improvement and Germplasm Innovation in Shanxi Province, Shanxi Agricultural University, Taiyuan, China; ^3^ Plant Protection College, Shenyang Agricultural University, Shenyang, Liaoning, China

**Keywords:** Soybean, SCN, CPT, GmPOD53L, POD, lignin

## Abstract

The soybean cyst nematode (*Heterodera glycines* Ichinohe, SCN) poses a significant threat to soybean yield, often leading to total crop failure in heavily infested areas. Identifying key resistance genes is essential for enhancing soybean resistance to SCN. This study demonstrates that pre-treatment with a bacterial extract, CPT, can enhance SCN resistance in soybean roots by increasing lignin content and peroxidase (POD) activity. Further investigation revealed that the Class III POD gene *GmPOD53L* (Glyma.02G171600) is upregulated under SCN stress, correlating with increased peroxidase activity and lignin content. Overexpression of *GmPOD53L* significantly bolstered SCN resistance, as evidenced by reduced SCN numbers, slowed SCN development, heightened lignin deposition, and elevated POD activity. Conversely, silencing *GmPOD53L* had the opposite effect. These findings suggest that *GmPOD53L* positively regulates SCN stress by enhancing lignin content and POD activity, thereby inhibiting SCN invasion and development. This study identifies *GmPOD53L* as a candidate gene for soybean breeding programs aimed at improving SCN resistance and provides a theoretical foundation for the development of related bio-based seed coatings and SCN-resistant breeding efforts.

## 1 Introduction

The soybean cyst nematode (*Heterodera glycines* Ichinohe, SCN) was first discovered by Russians in the northeastern region of China in 1899 and was later designated as the new species *Heterodera glycines* Ichinohe in 1952. The life cycle of the SCN can be summarized as follows: it hatches from the cyst as a second-stage juvenile (J2), seeks a host, infects it, forms a feeding site syncytium, develops, mates, and lays eggs. Soybean resistance to SCN primarily manifests in impeding the nematode’s infection, development within the root, and reproduction. This results in a slowdown or even cessation of the nematode’s growth and development, preventing it from completing its life cycle normally. However, the development of resistant varieties also has certain drawbacks. The continuous domestication of soybeans over a long period, coupled with strong selection in breeding to meet the traits required by modern agriculture, has directly or indirectly led to a reduction in genetic variation among modern cultivated varieties. As a result, soybean varieties resistant to SCN that have been bred from the cultivated soybean gene pool are increasingly losing their resistance to SCN due to the hybridization or shift between different SCN pathotypes (Kofsky et al., 2021). Moreover, the hybridization between SCN pathotypes and the continuous selection under resistance pressure have further accelerated this process (Chen et al., 2021; Chowdhury et al., 2021; Hua et al., 2018; Meinhardt et al., 2021).

Lignin, as a physical barrier, plays a key role in plant resistance to biotic stress, and its synthesis is regulated by Class III peroxidases. Lignin mainly deposited in the secondary walls of specific cell groups, giving plants sufficient mechanical strength and waterproof properties (Cesarino et al, 2016; Ranade et al., 2022; Sun et al., 2020). At the end of lignin synthesis, peroxidase (POD) and laccase (Lac) are usually used in secondary cell walls to interact with the three main types of monolignols (sinapyl alcohol, S unit; coniferyl alcohol, G unit and *p*-coumaryl alcohol, H unit) polymerization (Alejandro et al, 2012; Liu et al, 2011). In addition to the three main lignin units mentioned above, unconventional lignin units different from the three lignin units have been found in some plant groups in recent years, such as Tricin, a flavone, in grass (Lan et al, 2016), hydroxystilbenes in Poplar (*Populus trremula × alba*) tress (Cabané et al, 2004) and the highly modified monolignols in the Canary Island date palm (*Phoenix canariensis*) (Karlen et al, 2017). Plant cell wall is the first barrier against external hazards and are usually accompanied by increased lignin accumulation in response to various biological and abiotic stresses (Moura et al, 2010). Increased lignin accumulation can provide a basic barrier against pathogen transmission and reduce fungal enzymes and toxins infiltration into plant cell walls. Lignin-related compounds may cause fungi to lose their activity to infect the host and prevent pathogens from multiplying and moving (Miedes et al, 2014; Santiago et al, 2013).

Peroxidase is widely distributed in a variety of organisms, including animals, plants and microorganisms. At present, the most in-depth research is on non-animal peroxidase and further subdivided into three categories: Class I peroxidase, Class II peroxidase and Class III peroxidase. Among them, Class III peroxidase is also known as typical secretory plant peroxidase and form a large polygenic family in land plants (Blee et al, 2003; Cosio and Dunand, 2009; Luo et al., 2024). Class III peroxidase as one of important biotic or abiotic stress responsive enzymes, is able to respond positively to various stresses on plants (Kidwai et al, 2020). Meanwhile, Class III peroxidase is also involved in various stages of plant growth and development through combination with various substrates, such as seed germination (Amaya et al, 1999), cell wall metabolism (Francoz et al, 2015), fruit growth and development (Andrews et al, 2001), wound healing (Passardi et al, 2004), auxin metabolism (Kawano et al, 2001) and plant lignification (Barros et al, 2015). Some studies have shown that Class III peroxidase is related to polymerization in plants. For example, in tobacco and hybrid poplar, lignin levels are significantly reduced by antisense inhibition of *NtPrx60* and *PrxA3a*, respectively (Blee et al, 2003; Li et al, 2003). In *Arabidopsis thaliana*, analysis of mutants showed that loss of *AtPrx2* or *AtPrx25* function resulted in reduced lignin content (Shigeto et al, 2015).

Soybean (*Glycine max*) is one of the most important cash crops in the world, and it is the main source of vegetable protein and oil. But the yield of soybean is severely limited by SCN. There are more or less shortcomings in the traditional prevention and treatment works. Therefore, breeding resistant varieties has become a research hotspot. However, most of the commercial resistant varieties were derived from Peking and PI88788 (Mitchum, 2016). Continuous domestication and strong selection of target traits led to the decrease of genetic diversity, and consequently the resistance was gradually weakened (Kofsky et al, 2021). Biological control of SCN generally involves using natural enemies to reduce nematode infection numbers or delay their development through parasitism, competition for ecological niches, production of toxic substances, or induction of systemic resistance in plants. For instance, certain bacteria or fungi can parasitize the root regions of soybean plants to occupy infection sites or secrete nematocidal substances to directly kill nematodes, thereby achieving control objectives. Our previous studies have confirmed that the bacterium Sneb545 can induce resistance in soybeans against SCN and isolated a nematotoxic substances, cyclo (Pro-Tyr) (CPT), using semi-preparative high-performance liquid chromatography (HPLC) (Kang et al., 2018; Xing et al., 2020). However, the molecular mechanisms by which CPT induce resistance in soybeans against SCN remain unclear.

In this study, we found that CPT-coated treatment can induce resistance in soybean plants against the SCN by increasing the content of lignin and the activity of POD. We have confirmed that a POD gene, *GmPOD53L* (Glyma.02G171600), was able to respond positively to the stress of SCN. Meanwhile, overexpression of *GmPOD53L* mediated by *Agrobacterium rhizogenes* enhanced soybean resistance to SCN, whereas silencing of *GmPOD53L* via the Tobacco Rattle Virus (TRV)-Based Virus-Induced Gene Silencing (VIGS) method reduced soybean resistance to SCN, indicating that *GmPOD53L* plays a positive role in SCN stress response. The findings of this study offer a potential avenue for future soybean resistance breeding.

## 2 Materials and methods

### 2.1 Plant and nematode

A soybean susceptible variety, Williams 82 (W82), were grown at 27°C with a 12 h photoperiod in a greenhouse. Soybean cyst nematode (*Heterodera glycines* Ichnohe, SCN) race 3, one of the most widely distributed races in China, was tested in this study. SCN infested soil was mixed 1:1 with sterilized fine sand. Soybean seeds were sown to make SCNs grow and multiply. The cultivation lasted for two months, SCNs were separated from the infested soil, and the eggs were collected and incubated in Baermann Funnels at 27°C avoiding light. The hatched second stage juveniles (J2s) were collected daily and new distilled water was added. In order to ensure the activity of J2s, the collection process will be completed within one week. The isolated J2s SCN were utilized to infect various soybean materials.

### 2.2 CPT treatments

For CPT pretreatment, disinfected Williams 82 soybean seeds were coated with various concentrations (9.6 mmol·L^-1^, 4.8 mmol·L^-1^, 2.4 mmol·L^-1^, 1.2 mmol·L^-1^, 0.6 mmol·L^-1^, 0.3 mmol·L^-1^, 0.15 mmol·L^-1^, and 0.075 mmol·L^-1^) of CPT working solution and placed in plug trays to promote germination. Once the soybeans reached the two-leaf stage, they were transplanted into culture bowls of the same size for further growth. Each soybean plant was then inoculated with 1000 second-stage juveniles (J2) of the soybean cyst nematode. Fourteen days post-inoculation, nematode populations were statistically analyzed using acid fuchsin staining to determine the optimal concentration of CPT solution for inducing soybean resistance to SCN (Cook et al., 2012; Melito et al., 2010).

### 2.3 Quantitative RT- PCR

Total RNA was isolated from soybeans roots using the Total RNA Extraction Reagent (Vazyme, Nanjing, China), first-strand cDNA was synthesized using the PrimeScript™ RT reagent kit (TaKaRa, Beijing, China). qRT-PCR was performed to detect gene transcript levels using the 2X Universal SYBR Green Fast qPCR Mix (ABclonal, Wuhan, China) on a CFX96 qRT-PCR detection system (Bio-rad, San Francisco, CA, USA). Data were analyzed using the 2^-ΔΔCq^ method. Three groups of roots were sampled. Soybean *SKIP16* (Glyma.12G051100) served as reference genes. Primers used are detailed in **Supplementary Table S1**, and each data point was replicated three times.

### 2.4 Physiological index measurement

Detection of POD activity and lignin content determination were performed on a Multiskan GO (Thermo Scientific, Waltham, Massachusetts, USA) according to the manufacturer’s instructions with a Peroxidase Activity Assay Kit (Boxbio, Beijing, China) and a Lignin content detection kit, micromethod (Solarbio, Beijing, China). For each plant sample, a triplicate procedure was executed to ascertain the precision and uniformity of the experimental outcomes.

### 2.5 Transient overexpression in soybean roots

The *GmPOD53L* coding region was amplified using a 2×Phanta^®^ Max Master Mix (Vazyme, Nanjing, China) and ligated into the PRI101-GFP using a ClonExpress^®^ II One Step Cloning Kit (Vazyme, Nanjing, China). The constructs (OE) were transformed into *Agrobacterium rhizogenes* K599 Chemically Competent Cell (Protein Interaction, Wuhan, China), with the PRI101-GFP empty plasmid serving as a control (EV_1_). The 5-day-old soybean seedlings were obliquely cut off near the hypocotyl, a drop of bacterial mass was applied to the incision site and the seedlings were transplanted on wet vermiculite (Yang et al., 2021). Fluorescent hairy roots can be grown after 30 days of culture. The positive hairy roots were checked by a LUYOR-3415RG Hand-held Lamp (Luyor, Shanghai, China). In addition, qRT-PCR was used to detected the expression of target gene. The primers used were listed in **Supplementary Table S1**.

### 2.6 VIGS-mediated gene silencing in soybean roots

Virus-induced gene silencing was performed using pTRV to silence target genes in soybean roots. A 300-bp fragment of the 5’ cDNA of *GmPOD53L* was ligated into the pTRV2 vector. The constructed vectors were transferred into *Agrobacterium* EHA105 Chemically Competent Cell (Protein Interaction, Wuhan, China). Agrobacterium-mediated transformations were carried out as described previously (Chen et al., 2024). A 1:1 (v/v) mixture of pTRV1with pTRV2 (EV_2_) or pTRV2-GmPOD53L (KO) was injected around the roots of 4-day-old soybean plants at a rate of 5 mL per plant using a syringe. The process was repeated every 3 dpi, for a total of six applications. Samples were collected for gene expression analysis 4 days after the final inoculation. The primers used were listed in **Supplementary Table S1**.

### 2.7 Enrichment and inoculation of second stage juveniles

The suspensions of the second stage juveniles (J2s) were concentrated to 400pcs/ml through a 23μm sieve and then mixed 1:1 with a sterilized 0.2% water-agar to 200 pcs/mL (The final concentration of water-agar working solution is 0.1%). 5ml water-agar working solution was added into a 15ml centrifuge tube, and the roots of the plants were inserted into the bottom of the tube, so that the roots of the plants were completely immersed in the water-agar working solution (Each plant was inoculated with 1000 J2s). The outer wall of the centrifuge tube was wrapped with aluminum foil to ensure that the roots were protected from light and cultured in this state for two days. After two days, the plants were taken out, the water-agar working solution remaining in the roots was gently washed by running water, and then transferred to the sterilized vermiculite for further growth for 10 days.

### 2.8 SCN demographic assays

Under suitable conditions, SCNs can develop from J2 to adult in about 14 days at the earliest. If the culture time is too long, some cysts formed by female adult will fall off into the soil, increasing the statistical error. In this study, after 12 days post-inoculation (dpi), the roots were stained using the acid fuchsin method (Bybd et al, 1983). The total number of SCNs and the number of different development stages were counted (Cook et al, 2012).

### 2.9 Statistical analysis

Graphpad Prism 9.0 and Microsoft Excel 2019 were used for data statistics and graph analysis. Comparisons between two groups were conducted using the Student’s *t*-test. Significance levels are denoted as follows: *P < 0.05, **P < 0.01, ***P < 0.001, and ****P < 0.0001. Comparisons among multiple groups were performed using two-way ANOVA, with *P* < 0.01 considered significant. All values are presented as means ± standard deviation (SD) from at least three biological replicates.

## 3 Results

### 3.1 CPT coating treatment significantly increased the ratio of J2 SCN in soybean roots

**Figure 1:**
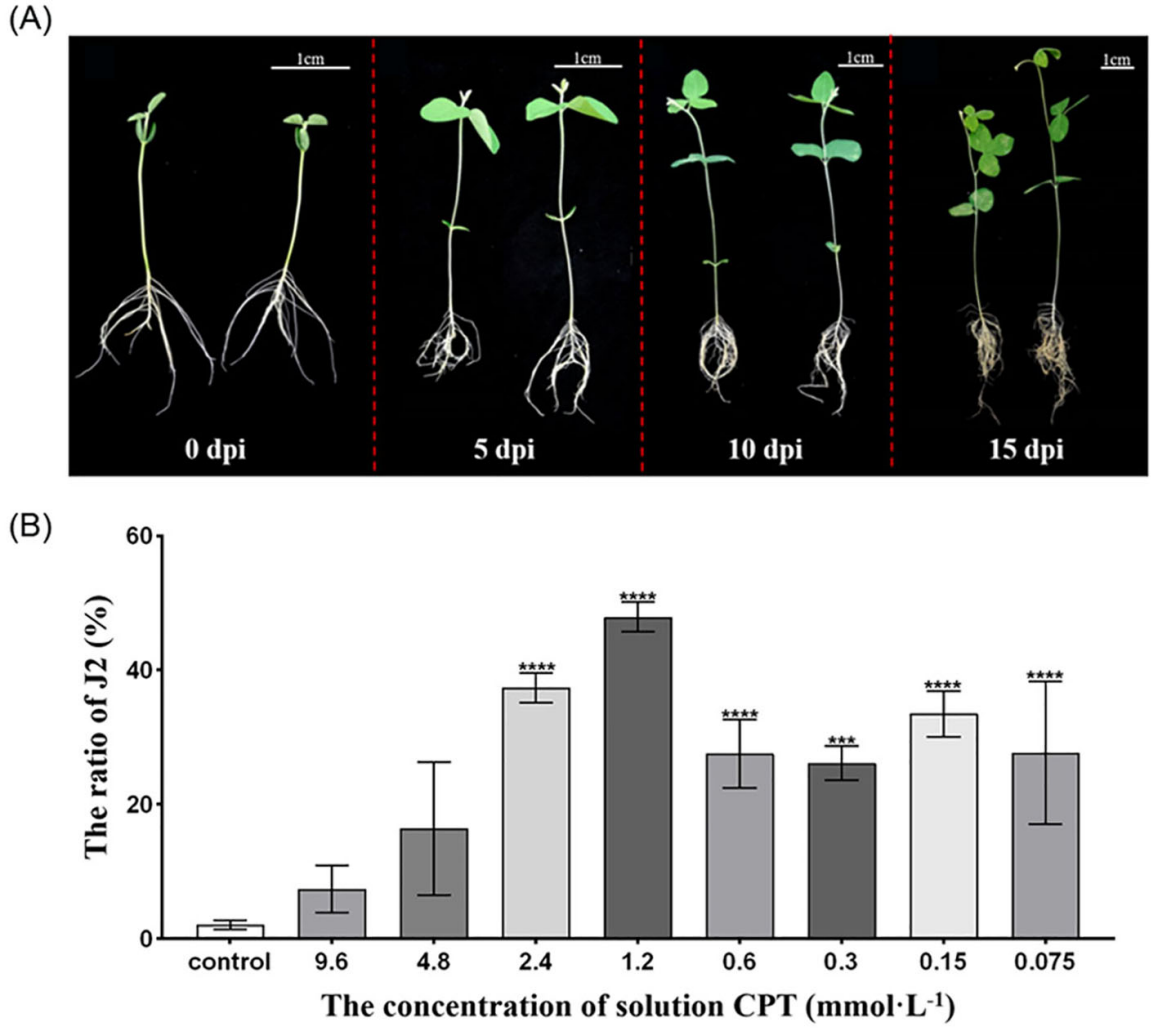
Effects of CPT treatment on soybean seedling development and J2 SCN Development in Roots. **(A)** Phenotypic comparison of soybean plant growth under CPT pretreatment. Left panel: Control group; Right panel: CPT-coated group. Bar=1 cm; **(B)** Proportion of J2 larvae in soybean roots coated with varying concentrations of CPT. Data are presented as means ± SD. Asterisks in the table denote statistical significance: ***(*p* < 0.001), and ****(*p* < 0.0001).

**Figure 2:**
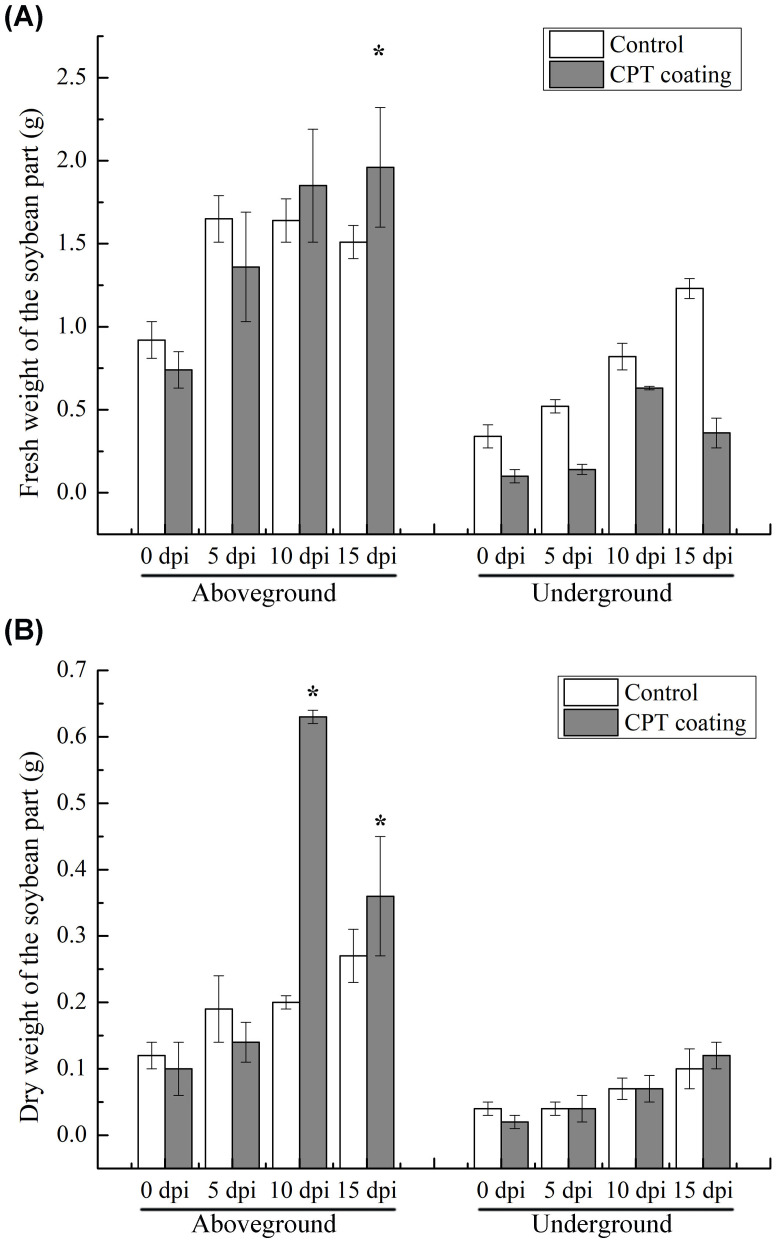
Determination of **(A)** fresh and **(B)** dry weight of soybean plants of control and CPT-coated groups. Dates are the mean ± standard error (n=5). *p* < 0.05 is considered statistically significant * (*p <*0.05).

Our previous research has found that CPT significantly reduces the number of nematodes in soybean roots (Kang et al., 2018; Xing et al., 2020). Here, we further investigated the effects of CPT pre-treatment on the growth and development of soybean plants, as well as its impact on SCN resistance in the roots systems. We first observations of the growth of soybean plants treated with CPT coating after reaching the two-leaf stage (7 day-old) revealed that CPT pre-treatment resulted in better growth compared to the control group (**Figure 1A**). Specifically, at 15 days post-inoculation (dpi), statistically significant differences (*p* < 0.05) were observed in the fresh (**Figure 2A**) and dry weights (**Figure 2B**) of the above-ground parts. However, no significant changes were detected in the fresh (**Figure 2A**) and dry weights (**Figure 2B**) of the below-ground parts.

The SCN life cycle involves hatching as J2 larvae, host penetration, syncytium formation, development, mating, and egg-laying. Soybean resistance to SCN mainly lies in impeding nematode infection, root development, and reproduction, thereby disrupting its normal life cycle. To further investigated the proportion of J2 of SCN in the root systems after CPT pretreatment. The plants were inoculated at the two-leaf stage, and the assessment was conducted 14 days post-inoculation (dpi). The results indicated that the proportion of J2 SCN in the roots was higher in all CPT-treated groups compared to the control group. Notably, the highest proportion of J2 was observed in plants treated with a CPT concentration of 1.2 mmol·L^-1^, suggesting that this concentration effectively arrested the development of SCN at the J2 stage (**Figure 1B**). The results indicate that CPT treatment effectively enhances the resistance of soybean roots to SCN and promotes the growth of the above-ground parts of soybean plants.

### 3.2 CPT pretreatment enhances soybean roots resistance under SCN stress

**Figure 3:**
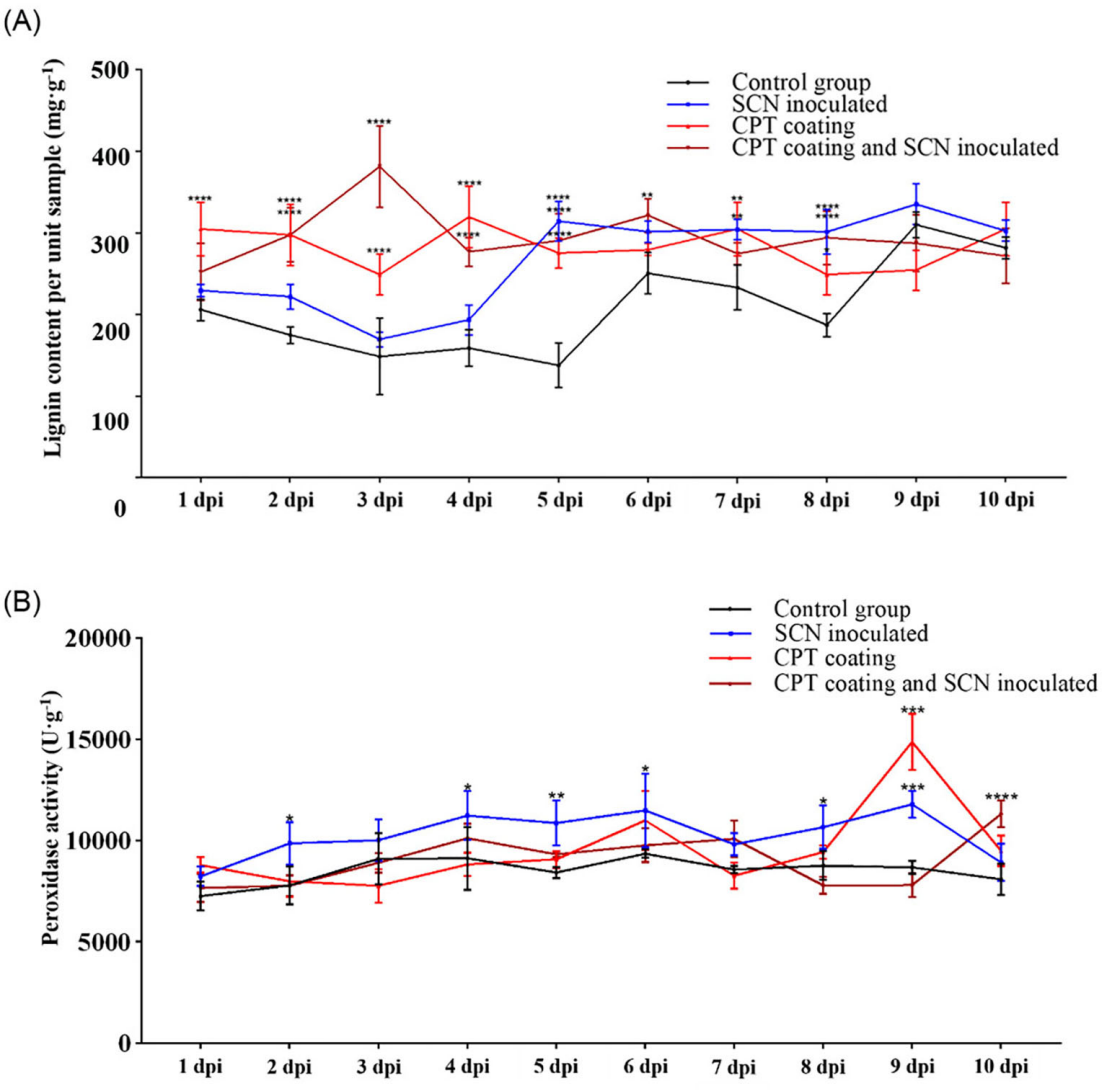
Physiological index detection of soybean roots under CPT treatment and/or SCN infection. **(A)** Lignin content and **(B)** POD activity in different treatments. The control group received no treatment. The SCN-inoculated group was infected with soybean cyst nematode (SCN) at the second leaf stage. The CPT-coating group was treated with CPT. The CPT-coating and SCN-inoculated group was infected with SCN at the second leaf stage after CPT treatment. Data are presented as means ± SD. Asterisks indicate statistical significance: *(*p* < 0.05), **(*p* < 0.01), ***(*p* < 0.001), and ****(*p* < 0.0001).

Under abiotic stress, plants produce more tightly bound lignin, which strengthens cell walls and supports normal cellular functions. From the perspective of physical defense, we measured the lignin content in soybean roots under CPT treatment after SCN infection. The lignin content in soybean roots increased significantly after SCN infection, with a notable rise at 4–5 dpi, coinciding with the formation of syncytia by the *H. Glycines*. This indicates that under SCN stress, the lignin synthesis pathway in soybean roots is activated to resist the stress. In contrast, the CPT-coated treatment group maintained a higher lignin content from 1 dpi onwards. CPT significantly enhances the overall resistance of soybean roots. When SCN infected plants pre-treated with CPT, lignin levels rose significantly as early as 3 dpi and remained high thereafter (**Figure 3A**). This suggests that CPT treatment can induce soybean plants to produce more lignin in the early stages of SCN infection, thereby enhancing the plant’s physical defenses against nematode invasion.

POD regulates lignin monomer polymerization downstream in the lignin synthesis pathway and modulates ROS levels by adjusting H_2_O_2_ production and scavenging under stress. We further measured the changes in POD activity in soybean roots under treatments of SCN infection, CPT coating, and SCN infection after CPT coating pretreatment. The POD activity in soybean roots increased after SCN infection compared to the control group, indicating that the roots’ POD activity is activated in response to SCN stress. There was no significant difference in POD activity between the CPT-coated treatment and the control group. However, in the combined CPT coating pretreatment and SCN infection, POD activity only showed a statistically significant difference at 10 dpi (**Figure 3B**). These results suggest that CPT coating pretreatment delays the induction of POD activity under SCN stress, indicating that POD can actively respond to SCN. Additionally, the enhanced resistance of soybean plants after CPT coating means that lower POD activity is sufficient to defend against nematodes, thereby reducing damage caused by oxidative stress.

These findings presented above indicate that CPT (a specific treatment or compound, as contextually relevant) has a significant regulatory effect on both the lignin content and POD activity within the soybean root system. This dual-action mechanism of CPT not only enhances the structural integrity of the soybean roots thereby providing a robust defense mechanism against these harmful pathogens.

### 3.3 Overexpression of *GmPOD53L* enhances soybean roots resistance to *H. Glycines*

**Figure 4:**
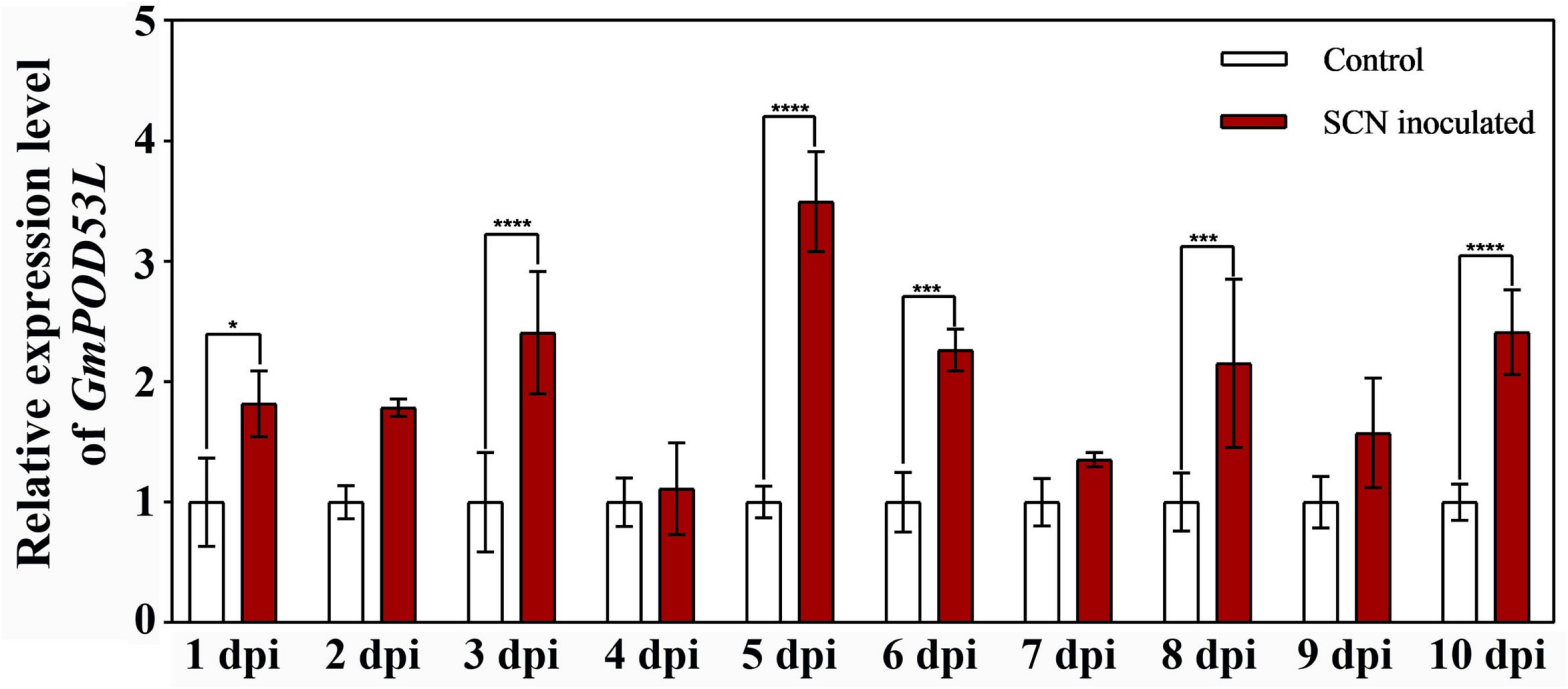
*GmPOD53L* positively responded to SCN stress. Relative expression levels of *GmPOD53L* in soybean (n=3). Data are means ± SD, Statistical significance is indicated as follows: *p* < 0.05 (*), *p* < 0.001 (***), and *p* < 0.0001 (****).

Previous research through combined transcriptomic and metabolomic analyses confirmed that CPT treatment enhances the activity of POD and the expression of several POD family genes (Kang et al., 2018). We further investigated the role of Class III POD gene, *GmPOD53L*, in regulating soybean resistance to SCN. qPCR-PCR revealed that *GmPOD53L* is significantly upregulated after SCN infection, with the highest expression level observed at 5 dpi, indicating that *GmPOD53L* can respond to SCN stress (**Figure 4**).

**Figure 5:**
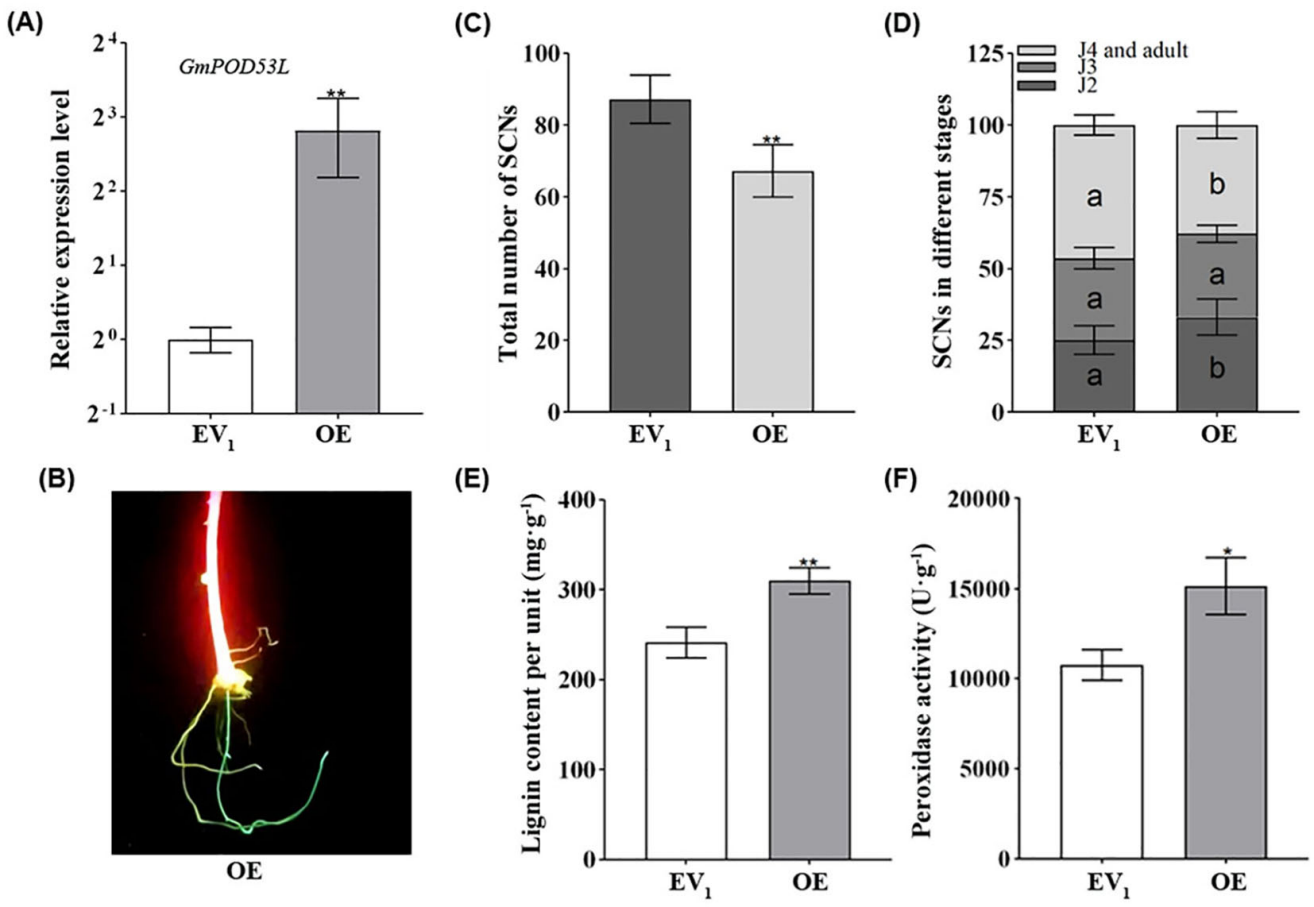
Overexpression of *GmPOD53L* enhances soybean roots resistance against *H*. *Glycines* by increasing the peroxidase activity and lignin content. **(A)** Relative expression levels of *GmPOD53L* in over-expressed groups (OE, n=6) and empty vector control groups (EV_1_, n=3) (Student’s *t*-test, *p* < 0.01); **(B)** Fluorescent hairy roots (OE) can be easily distinguished with LUYOR-3415RG used as the excitation light source. **(C)** Total number of SCNs in soybean roots (p< 0.01) **(D)** Demographics assays of SCNs in different stages between over-expressed groups (OE, n=5) and empty vector control groups (EV_1_, n=5) (one-way ANOVA, in EV_1_ versus OE, a and b mean that the dates were statistical different, *p <*0.05); **(E)** Lignin content in over-expressed groups (OE, n=3) and empty vector control groups (EV_1_, n=3) (Student’s *t*-test, *p* < 0.01); **(F)** Peroxidase activity in over-expressed groups (OE, n=3) and empty vector control groups (EV_1_ n=3) (Student’s *t*-test, *p* < 0.05).

To further investigated the function of *GmPOD53L* in regulation soybean roots resistance to SCN. The overexpression of *GmPOD53L* was achieved by *Agrobacterium rhizogenes* inducing hairy roots. qPCR was employed to detect the expression level of *GmPOD53L* in hairy roots. In the overexpression (OE) groups, the relative expression level of *GmPOD53L* was significantly higher than that of the empty vector (EV_1_) control, ranging from 3- to 7-fold (**Figure 5A**). Moreover, laser detection of GFP fluorescence confirmed the successful transformation of the cultured soybean hairy roots, as evidenced by the distinct presence of positive GFP signals (**Figure 5B**). The transformed hairy roots were inoculated with SCN, and at 12 dpi, the roots were stained using the acid fuchsin method. A significant statistical difference was observed between the OE groups and the EV_1_ control in terms of the total number of nematodes. In the OE groups, the total number of SCNs was lower than that in the EV_1_ control (**Figure 5C**). When comparing the number of SCNs at the same developmental stage between the OE groups and the EV_1_ control, significant statistical differences were found in the number of J2s, J4s, and adults. Specifically, the number of J2s was significantly higher in the OE groups, while the proportions of J4s and adults were significantly lower (**Figure 5D**).

To determine whether overexpression of *GmPOD53L* would lead to alterations in downstream products and affect resistance of soybean roots to SCN, lignin content and POD activity were measured. At 5 dpi with SCN, both lignin content (**Figure 5E**) and POD activity (**Figure 5F**) were found to be elevated in the overexpression (OE) groups compared to the empty vector (EV_1_) control. These results demonstrate that overexpression of *GmPOD53L* can significantly enhance the resistance of soybean roots to SCN invasion, thereby retarding SCN development and conferring additional resistance to SCN.

### 3.4 Silencing of *GmPOD53L* weakens soybean roots resistance to *H. Glycines*

**Figure 6:**
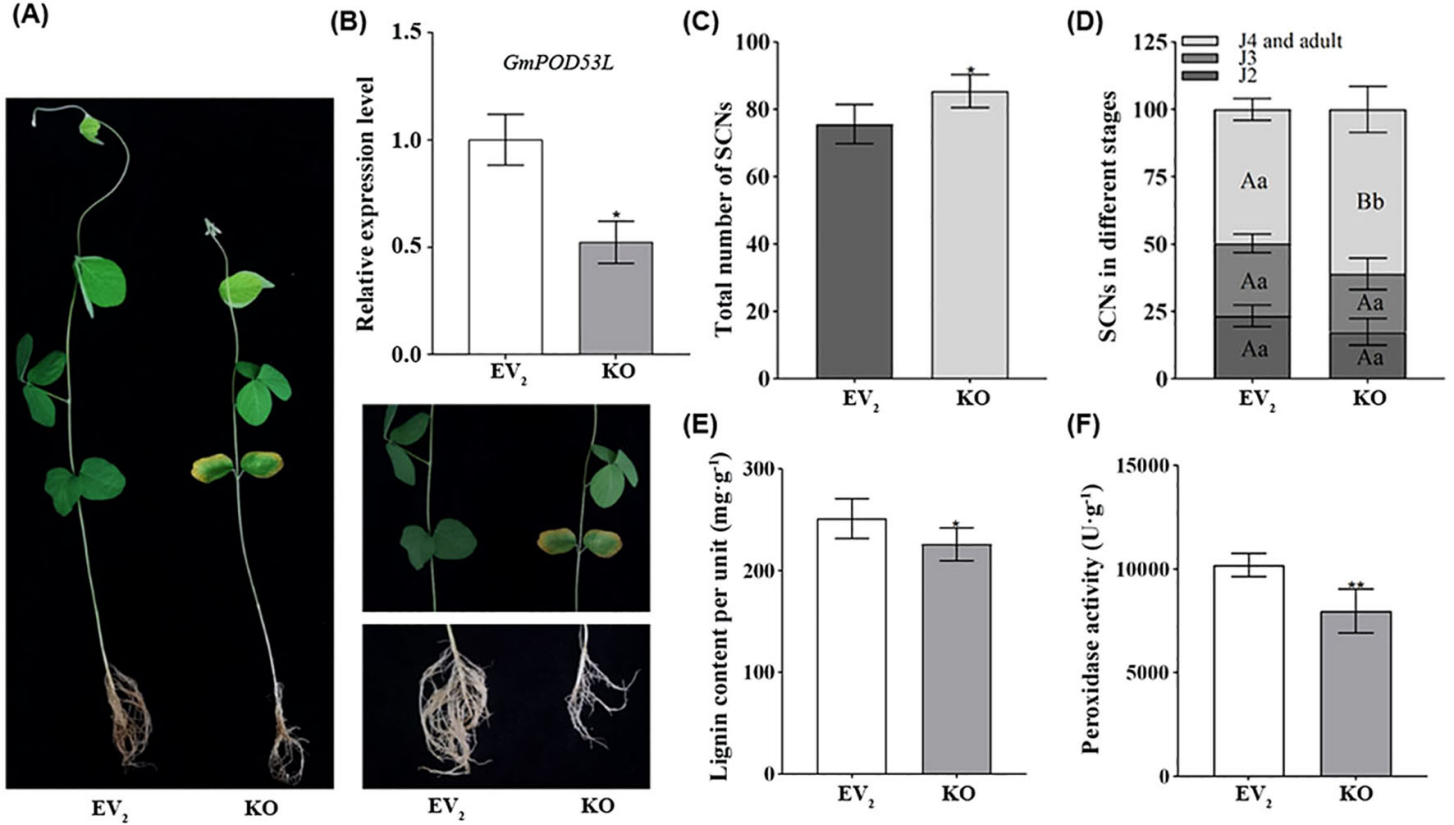
Silencing of *GmPOD53L* weakens soybean roots resistance against *H*. *Glycines* by reducing the peroxidase activity and lignin content. **(A)** The induced soybeans (right) grew significantly slower than the normally grown soybeans (left). The screenshot displayed viral symptoms in the leaves and underdeveloped root system in the inoculated plants; **(B)** Relative expression levels of *GmPOD53L* in pTRV1/pTRV2: GmPOD53L groups (KO, n=6) and empty vector control groups (EV_2_, n=3) (Student’s *t*-test, *p* < 0.05); **(C)** Demographics assays of SCNs in pTRV1/pTRV2: GmPOD53L groups (KO, n=5) and empty vector control groups (EV_2_, n=5), (Student’s *t*-test, *p*<0.05); **(D)** Demographics assays of SCNs in different stages between pTRV1/pTRV2: GmPOD53L groups (KO, n=5) and empty vector control groups (EV_2_, n=5) (one-way ANOVA, a and b mean that the dates were statistical difference, *p <*0.05, A and B mean that the dates were significant statistical difference, p<0.01). **(E)** Peroxidase activity in pTRV1/pTRV2: GmPOD53L groups (KO, n=6) and empty vector control groups (EV_2_, n=3) (Student’s *t*-test, *p* < 0.05); **(F)** Lignin content in pTRV1/pTRV2: GmPOD53L groups (KO, n=6) and empty vector control groups (EV_1_, n=3) (Student’s *t*-test, *p* < 0.01).

The silencing of *GmPOD53L* was accomplished through Tobacco Rattle Virus (TRV)-mediated Virus-Induced Gene Silencing (VIGS). Compared with the normally growing plants, those subjected to VIGS exhibited significantly delayed development, characterized by varying degrees of mosaic patterns, chlorosis, and underdeveloped root systems (**Figure 6A**). The silencing of *GmPOD53L* was detected by qPCR. In pTRV1/pTRV2: GmPOD53L groups (KO), the relative expression level of the *GmPOD53L* gene was significantly downregulated, with a reduction of more than 50% compared to the EV_2_ (**Figure 6B**).

The KO mutant lines were inoculated with SCN, and at 12 dpi, the roots were stained using the acid fuchsin method. Subsequently, the total number of SCNs and the number of nematodes at different developmental stages were counted. A significant statistical difference was observed between the KO groups and the EV_2_ control group in terms of the total number of nematodes. Specifically, the total number of SCNs was higher in the KO groups than in the EV_2_ groups (**Figure 6C**). Notably, the proportion of J2s was lower in the KO groups, whereas the proportions of J4s and adults were significantly higher than in the EV_2_ control group (**Figure 6D**). Meanwhile, the POD activity and lignin content were measured in the KO lines. In the KO groups, both lignin content (**Figure 6E**) and POD activity (**Figure 6F**) and were found to be lower than in the EV_2_ control. These results collectively suggest that silencing of *GmPOD53L* compromises soybean’s resistance to SCN, thereby facilitating increased SCN invasion.

## 4 Discussion

Lignin serves as the primary physical defense barrier against biotic stress in plants, playing a crucial role in resisting pathogen invasion and restricting pathogen movement within the plant (Ithal et al., 2007). Under biotic or abiotic stress, the expression of lignin synthesis-related genes and the amount of lignin deposition increase correspondingly. Different types of stress can also lead to changes in lignin composition. For example, under abiotic stress conditions such as ozone exposure, high nitrogen stress, mechanical damage, and osmotic stress, it has been proven to induce angiosperms and gymnosperms to produce more tightly bound lignin, with a higher proportion of C-C bonds and H units detected in stress lignin (Cabané et al., 2004; Pitre et al., 2007; Sato et al., 2010). In addition to acting as a physical barrier against pathogen spread under biotic stress, unpolymerized lignin units also exhibit certain antibacterial activities (Barber et al., 2000). The S unit plays a particularly prominent role in plant resistance to biotic stress. Plants with a higher proportion of S units in lignin show stronger resistance to pathogen infection (Gallego-Giraldo et al., 2018; Wuyts et al., 2006). Our prior research, which assessed the lignin content in both SCN-resistant and susceptible soybean cultivars, revealed that resistant cultivars typically exhibit higher lignin content compared to their susceptible counterparts.

Lignin synthesis is regulated by Class III peroxidases, which play a crucial role in plant growth and development, participating in a wide range of physiological responses, particularly in response to various biotic and abiotic stresses (Zheng et al., 2023). As an important enzyme involved in biotic stress responses, Class III peroxidases have been shown to be involved in ROS production and to trigger the microbe-associated molecular patterns (MAMPs) pathway, thereby activating plant immunity (Daudi et al., 2012; O’Brien et al., 2012). Numerous studies have shown that the accumulation of Class III peroxidases increases following pathogen infection, working in concert with NADPH oxidase to induce H_2_O_2_ production during the early stages of pathogen response, leading to oxidative stress (Choi et al., 2007; Wally and Punja, 2010). Meanwhile, several studies have also clarified that Class III peroxidases regulate the polymerization of lignin units downstream of the lignin synthesis pathway under biotic or abiotic stress (Bonawitz and Chapple, 2010).

The POD genes have been identified as key players in numerous physiological and developmental processes, such as cell elongation, cross-linking of cell wall components, lignin and suberin biosynthesis, ROS scavenging, wound healing, phytoalexin production, and defense mechanisms against both biotic and abiotic stresses (Aleem et al., 2022; Daudi et al., 2012; Jiao et al., 2024). In *Arabidopsis thaliana*, peroxidase gene *AtPrx64* overexpression exhibited enhanced tolerance to aluminum stress (Wu et al., 2017). The cold-inducible gene *AtPrx3* encodes a POD that confers increased resistance to salt and drought stresses (Llorente et al., 2002). Ectopic overexpression of *POD* genes resulted in improved germination under cold, salt, and dehydration stresses (Kumar et al., 2012). In tomato, down-regulation of the *POD* gene was associated with reduced susceptibility to bacterial speck (Coego et al., 2005). Overexpression of rice *OsPrx114* gene in transgenic carrot plants led to increased resistance to fungal diseases (Wally and Punja, 2010). Similarly, overexpression of *GsPRX9* in soybean composite seedlings resulted in enhanced tolerance to salt stress (Jin et al., 2019). Conversely, knockout lines of pepper for the extracellular gene *CaPO2* showed increased vulnerability to bacterial pathogens, while overexpression of *CaPO2* enhanced resistance to bacterial pathogens (Choi et al., 2007). Our research indicates that CPT treatment can activate POD activity and the expression of POD genes, yet the precise regulatory mechanisms, such as the involvement of specific signaling pathways and transcription factor binding sites, remain to be elucidated.

Our study revealed that *GmPOD53L* is a key player in the soybean’s response to SCN stress. Under SCN stress, *GmPOD53L* expression is significantly upregulated. Transgenic experiments further demonstrated that overexpression of *GmPOD53L* not only increases POD activity and lignin content but also enhances resistance to SCN invasion and retards SCN development, thereby conferring additional resistance to SCN. Conversely, downregulation of *GmPOD53L* expression resulted in the opposite effects. *GmPOD53L* operates downstream in the lignin synthesis pathway, primarily regulating lignin deposition by influencing the polymerization of lignin units (Bonawitz and Chapple, 2010). Class III peroxidases have also been implicated in immune responses against pathogenic bacteria and plant-parasitic nematodes (Jin et al., 2011). Many studies have shown that the accumulation of Class III peroxidase increases after pathogen infection, and together with NADPH oxidase, induces H_2_O_2_ production during early pathogen response, leading to oxidative stress (Choi et al, 2007; Wally and Punja, 2010). In this study, by modulating *GmPOD53L* expression, we observed changes in POD activity and lignin content in roots under SCN stress exposure, which may underlie the observed differences in nematode numbers. Based on the aforementioned findings, it is evident that *GmPOD53L* enhances POD activity and lignin content, thereby bolstering resistance to SCN stress. However, the regulation of *GmPOD53L* by salicylic acid (SA) and jasmonic acid (JA) signaling pathways remains a critical area requiring further investigation. For instance, the synergistic action of *GmPOD53L* with other resistance genes could enhance the overall defense response, potentially through the reinforcement of cell wall structures or the activation of additional defense pathways. Conversely, *GmPOD53L* may also exert independent effects, contributing to resistance through unique mechanisms such as the modulation of oxidative stress responses. Moreover, the functions of other Class III POD genes in soybean resistance to SCN are also key areas that need to be uncovered in the future.

## 5 Conclusions

The results of this study demonstrated that a cyclic dipeptide extract from bacteria, known as CPT, can increase the number of J2-stage SCN, impede further SCN development, and promote biomass accumulation in the above-ground parts of soybean plants. Additionally, CPT treatment enhances POD activity and lignin content in soybeans, thereby improving overall plant resistance. Further functional validation experiments on the CPT-activated *GmPOD53L* gene revealed that overexpression of *GmPOD53L* significantly strengthens soybean root resistance to SCN stress, with increased POD activity and lignin content, conferring additional resistance to SCN. Conversely, silencing of *GmPOD53L* led to decreased POD activity and lignin content, resulting in reduced resistance to SCN in soybeans.

## Data Availability

The datasets presented in this study can be found in online repositories. The names of the repository/repositories and accession number(s) can be found in the article/**Supplementary Material**.

## Supplementary material

Supplementary Table 1 List of primers used in this study.
